# Making sense of healthy and sustainable food: adolescents’ voices on what it means, why it matters, and future change

**DOI:** 10.1093/heapro/daaf230

**Published:** 2026-01-31

**Authors:** Anouk Mesch, Femke Hoefnagels, Judith Gulikers, Renate Wesselink, Laura H H Winkens, Sanne Raghoebar, Annemien Haveman-Nies

**Affiliations:** Education & Learning Sciences, Wageningen University & Research, Hollandseweg 1, Wageningen 6706 KN, The Netherlands; Consumption & Healthy Lifestyles, Wageningen University & Research, Hollandseweg 1, Wageningen 6706 KN, The Netherlands; Consumption & Healthy Lifestyles, Wageningen University & Research, Hollandseweg 1, Wageningen 6706 KN, The Netherlands; Department of Nutrition and Health, Louis Bolk Institute, Kosterijland 5, Bunnik 3981 AJ, The Netherlands; Education & Learning Sciences, Wageningen University & Research, Hollandseweg 1, Wageningen 6706 KN, The Netherlands; Education & Learning Sciences, Wageningen University & Research, Hollandseweg 1, Wageningen 6706 KN, The Netherlands; Consumption & Healthy Lifestyles, Wageningen University & Research, Hollandseweg 1, Wageningen 6706 KN, The Netherlands; Consumption & Healthy Lifestyles, Wageningen University & Research, Hollandseweg 1, Wageningen 6706 KN, The Netherlands; Consumption & Healthy Lifestyles, Wageningen University & Research, Hollandseweg 1, Wageningen 6706 KN, The Netherlands; Academic Collaborative Centre AGORA, GGD Noord- en Oost-Gelderland, Rijksstraatweg 65, Warnsveld 7231 AC, The Netherlands

**Keywords:** secondary schools, adolescence, food choices, interventions, participation, mixed methods

## Abstract

Despite growing research on school food interventions aimed at promoting sustainable and healthy diets, the perspectives of adolescents regarding those interventions remain underexplored. This study explores adolescents’ understanding, perceived importance, and proposed strategies for (facilitating) healthy and sustainable food choices. A mixed-methods study was conducted among 296 adolescents (aged 12–16) at four Dutch secondary schools. Data was gathered through four consecutive methods: a questionnaire, focus group discussions, classroom discussions, and a group poster assignment. Quantitative data were analysed using descriptive statistics and ordinal logistic regression analyses. Qualitative data were analysed inductively through thematic analysis. Adolescents primarily associated healthy and sustainable food with vegetables, fruit, and organic products. Two-thirds of the sample perceived eating healthy food as (very) important, compared to 21% for sustainable food, while 12% indicated not knowing what sustainable food entails. Proposed strategies to facilitate healthy and sustainable food were grouped in four main categories: ‘strategies to change the food environment’ (e.g. price), ‘strategies to change the food system’ (e.g. sustainable food production), ‘strategies for communication and social support’ (e.g. advertisements), and ‘individual behaviour change strategies’ (e.g. grocery planning). Most strategies targeted the food environment and/or the food system. While health aspects of food were well understood and perceived as important by adolescents, future approaches should emphasize the relevance of sustainable food for adolescents by addressing values they care about. Adolescents call for structural changes, particularly requiring governmental and organizational actions to improve the offer of healthy, sustainable, and affordable food, requiring collaboration of diverse stakeholders.

Contribution to Health Promotion

- Schools provide an important setting to encourage dietary behaviours that support both individual health and planetary health.
- Until now, the perspectives of adolescents on healthy and sustainable food, and on potential interventions, have received limited attention.
- Adolescents demonstrate a solid understanding of healthy food and consider it important; they are also to some extent familiar with the concept of sustainable food.
- Adolescents primarily call for changes in the broader food environment and food system, emphasizing the need for governmental and organizational actions to ensure that healthy, sustainable, and affordable food is more accessible.

## Introduction

Healthy and sustainable dietary patterns are defined by the FAO and WHO as ‘dietary patterns that promote all dimensions of individuals’ health and wellbeing, have low environmental pressure and impact, are accessible, affordable, safe and equitable, and are culturally acceptable’ (p. 9) (Food and Agriculture Organisation and World Health Organisation 2019). However, globally, dietary patterns have shifted towards more unhealthy and unsustainable patterns, contributing to major public health issues, such as diet-related chronic diseases, as well as major environmental issues, such as an increase in greenhouse gas (GHG) emissions (Willett *et al*. 2019). The consumption of foods that pose a risk to individual and planetary health has increased, among which are foods high in calories, ultraprocessed foods, and high amounts of animal-source foods (Willett *et al*. 2019). A recent report by the WHO shows that globally, many adolescents do not adhere to healthy and sustainable dietary patterns. To illustrate, 38% of adolescents indicated to eat fruit every day, similar to the number of adolescents reported to eat vegetables on a daily basis (38%) (Badura *et al*. 2024). Given that dietary behaviours are largely shaped before adulthood, these numbers highlight the need for strategies that facilitate healthy and sustainable dietary behaviours among youth.

Adolescence is a unique transition period between childhood and adulthood. During this time in life, individuals start to gain a sense of autonomy, develop their identity, and increasingly start making their own dietary choices (Stok *et al*. 2010, De Vet *et al*. 2014, Raghoebar *et al*. 2024). Considering this time of growing autonomy and development, it is essential to understand what adolescents ‘themselves’ think about healthy and sustainable food, to be able to connect strategies to their lived experiences. While adolescents generally demonstrate a solid understanding of healthy food, this knowledge often fails to translate into healthy food choices (Ronto *et al*. 2016, Damen and Steenbekkers 2022, Lanham and Van der Pols 2025). Considering sustainability, there are indications for increased awareness of environmental issues among adolescents, e.g. demonstrated by youth climate movements like ‘Fridays for Future’ (Thompson *et al*. 2021). Nevertheless, previous literature identified a dip in sustainability consciousness in adolescence compared to earlier childhood as well as adulthood (Olsson and Gericke 2016). A recent scoping review by Lanham and Van der Pols (2025) showed that adolescents indeed have a limited understanding of sustainable diets, leading to low consideration of sustainability and low aspiration to make sustainable food choices (Lanham and Van der Pols 2025). Moreover, only a few previous studies have captured adolescents’ understanding of the combination of healthy and sustainable diets (Lanham and Van der Pols 2025). Moreover, adolescents’ ability to act on values of health and sustainability varies depending on the context and level of autonomy they experience (Lanham and Van der Pols 2025). To illustrate, adolescents may experience more autonomy in food choices out of home and at school (depending on whether schools provide meals) and can then make their own decisions regarding healthy and sustainable food. Given this growing autonomy, it is interesting to gain insight into whether adolescents ‘themselves’ understand and consider aspects of health and sustainability when making autonomous food choices, in order to identify possible entries to promote healthy and sustainable food in this age group.

To facilitate healthy and sustainable dietary behaviour among adolescents, there has been increasing interest in food and nutrition interventions in and around secondary schools as schools have the potential to target a wide and diverse group of adolescents within a structured learning environment (Capper *et al*. 2022). Nevertheless, it is still unclear what type of intervention strategies have the potential to result in long-term dietary change (Lanham and Van der Pols 2025). Many school-based interventions have mainly been effective in targeting individual determinants, such as knowledge and attitudes, but have hardly resulted in long-term dietary change (Lanham and Van der Pols 2025, Mesch *et al*. 2025a). According to Yeager *et al*. (2018), more traditional preventive classroom interventions that rely mainly on increasing knowledge (related to various topics such as unhealthy snacking and bullying) have not yielded the desired effects among adolescents, presumably as they fail to align with adolescents’ interests and direct needs for autonomy and social support (Yeager *et al*. 2018). Given adolescents’ curiosity, creativity, growing autonomy, and desire to have their voices heard, research has increasingly emphasized the importance of actively engaging adolescents in food and nutrition research and education, such as through cocreation approaches. For instance, there have been calls to explore adolescents’ perspectives on their food environment (Kelly *et al*. 2021), on their food experiences at school (Browne *et al*. 2020), or to use the opinions and lived experiences of students to develop future food and nutrition interventions (Rose *et al*. 2021). Additionally, it is essential to engage a range of adolescents from diverse contexts (e.g. geographical location and educational track), as their needs may differ due to contextual differences. For example, differences in their opinions and lived experiences may be influenced by differences in their (urban or rural) food environments or by educational differences (De Clercq *et al*. 2017, Egg *et al*. 2020, Franca *et al*. 2022). Hence, in order to develop intervention strategies that resonate with the needs and developmental stage of adolescents in diverse environments, it is essential to understand what adolescents themselves propose regarding intervention strategies to facilitate healthy and sustainable diets.

The current study therefore aims to explore adolescents’ perspectives on (promoting) healthy and sustainable food, in terms of their understanding, perceived importance, and suggestions for strategies to support healthy and sustainable food during adolescence. This mixed-methods study is part of the ‘SWITCH’ project, which—through a socioecological lens—aims to understand how we can empower adolescents at secondary schools to engage in healthier and more sustainable dietary behaviours. Understanding adolescents’ perspectives on this topic can inform future strategies promoting healthy and sustainable food in and around schools. The following questions guided this research:

‘RQ1. What are adolescents’ perspectives on (facilitating) healthy and sustainable food, in terms of understanding, perceived importance, and proposed strategies to facilitate healthy and sustainable dietary behaviours?’

 ‘RQ1.1 What differences can be observed, based on geographical location and educational track, in terms of understanding, perceived importance, and proposed strategies?’

## Materials and methods

### Study design

**Figure 1:**
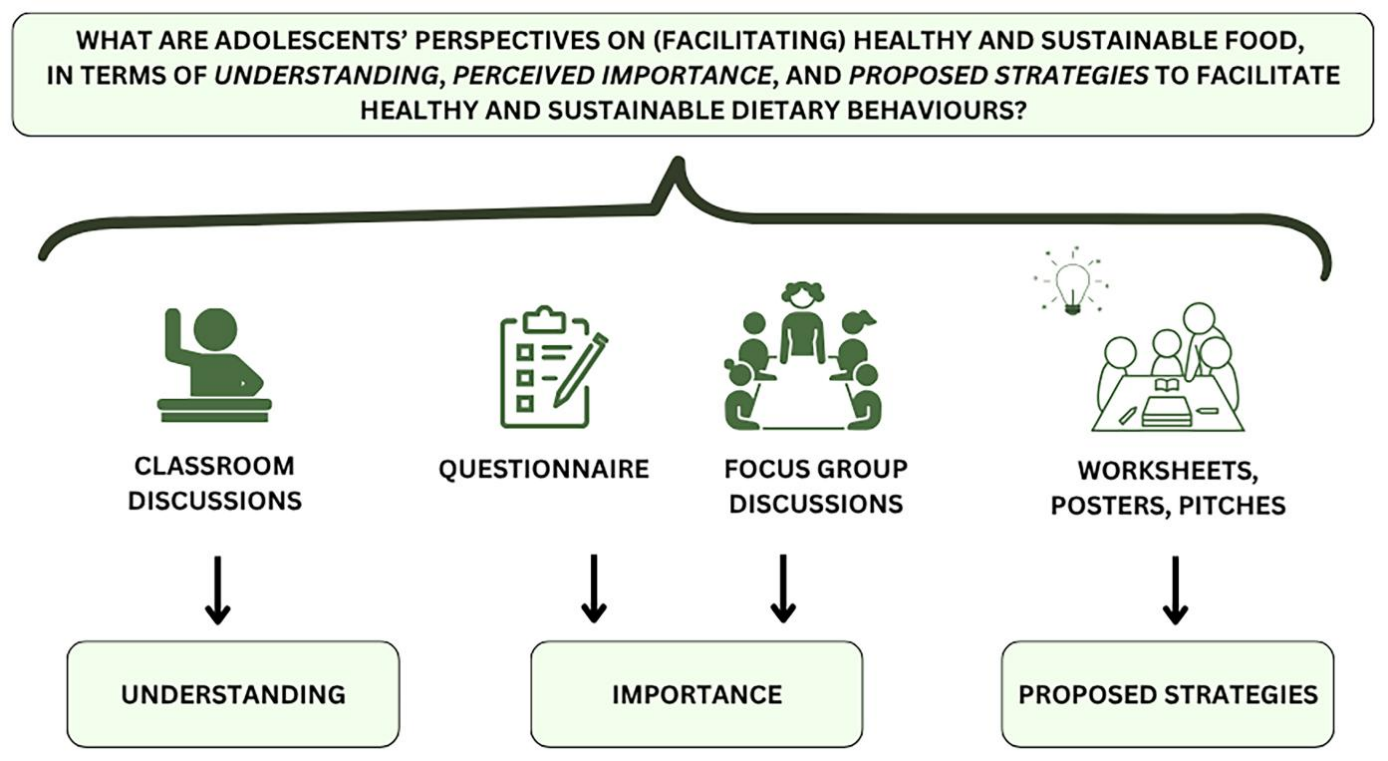
Overview of the used methods and their contribution to the different aspects of the research question.

This study followed a mixed-methods design, using four consecutive, diverse methods: (i) a questionnaire, (ii) focus group discussions, (iii) a classroom discussion, and (iv) a group poster assignment. Data was collected at secondary schools during two sessions, each lasting 45–60 minutes. These methods were chosen in order to address multiple aspects of our research question, regarding students’ understanding and perceived importance of healthy and sustainable food, as well as what strategies they proposed to facilitate healthy and sustainable food. An overview of which methods contributed to what aspect of the research question can be found in Fig. 1. Moreover, these sessions were designed in collaboration with (student) teachers for both data collection and educational purposes. Specifically, we consulted student teachers (*n*  *=*  *6*) at Wageningen University & Research for a brainstorm session to develop the first concept of the study design, to ensure that the design and study materials were tailored to adolescents in the secondary school context. The research team then further designed the methods and materials, which were ultimately checked by two teachers from the participating schools (feedback by email) and further discussed (in person) with one secondary school teacher not participating in the study. Through this procedure, the study materials also resulted in educational material on healthy and sustainable food, which is now available (open source) to be independently implemented by teachers (https://osf.io/42h8b/).

### Participants and recruitment

In total, 296 adolescents from four Dutch secondary schools participated in the study. Via the research network of the team, we selected schools through purposive sampling in order to include schools that were diverse in location and educational track. Two schools were located in the East of the Netherlands, in low urbanized municipalities (27 244 inhabitants and 780 addresses per km^2^) or moderately urbanized municipalities (59 195 inhabitants and 1163 addresses per km^2^). The other two schools were located in the West of the Netherlands, in very urbanized municipalities (663 900 inhabitants and 4058 addresses per km^2^; 918 117 inhabitants and 6075 addresses per km^2^) (Centraal Bureau voor de Statistiek 2025). For this study, we categorized low and moderately urbanized municipalities as ‘rural’ and very urbanized municipalities as ‘urban’. To categorize educational track, we used the three tracks of secondary education in the Netherlands: prevocational secondary education (‘vmbo’, 4 years of schooling), senior general secondary education (‘havo’, 5 years of schooling), and preuniversity education (‘vwo’, 6 years of schooling). Location, educational track, and further characteristics of the participating schools can be found in Supplementary Table S1.

To recruit schools, we first contacted teachers by email and/or phone to inform them about the aim of the study and the content of the two sessions. Together with the teachers, we agreed on the subject in which the study could best be integrated and the number of participating classes per school. This resulted in 13 school classes in four schools. Consent was obtained from both adolescents and their caregivers. Before the start of the study, caregivers received a (digital) letter via teachers to inform them about the study activities through which they had the option to have their child opt out of the study (passive consent). At the start of the first session, we informed adolescents about the purpose and procedure of the study and asked them for active written informed consent for the use of their personal data. We presented the two sessions to adolescents as guest lessons, and consequently, all students in class participated in the research activities as learning activities. Nevertheless, in case they or their caregivers did not give consent for the use of their data, their individual data was omitted directly after data collection (*n*  *=* 13). This study was approved by the Social Sciences Ethical Committee (SEC) at Wageningen University & Research (approval date: 20 February 2023).

### Procedure and materials

Between May and December 2023, two researchers (A.M. and F.H.) and a research assistant collected data at four schools. The procedure is described per data collection method below.

#### Questionnaire: demographics and importance of healthy and sustainable food

At the start of the first session, students filled out a questionnaire that contained questions on demographics, dietary restrictions, money expenditure on food, and perceived importance of eating healthy and sustainable food. Concerning dietary restrictions, students filled out whether they followed any cultural prescriptions, a pescetarian, vegetarian, or vegan diet, had any allergies or intolerances, or had other dietary restrictions. Additionally, for money expenditure, students reported how often they (on average) spend their own money on food, on a five-item scale ranging from ‘(almost) never’ to ‘every school day’. Regarding the importance of healthy and sustainable food, students filled out two items on how important it is for them to eat (i) healthy food and (ii) sustainable food, on a five-item scale ranging from ‘not important at all’ to ‘very important’, with an additional option ‘I do not know what healthy/sustainable food entails’. An English translation of the questionnaire can be found in Supplementary Materials S2.

#### Focus groups: importance of healthy and sustainable food

In consultation with teachers, students were grouped into focus groups of five to eight students, facilitated by one of the researchers (A.M. or F.H.) or research assistant. In some classes, there were more groups than facilitators, so the remaining group(s) were teacher-led (*n*  *=* 1) or student-led (*n*  *=* 7). In sum, 50 focus groups were conducted and audio-recorded and lasted ∼25 minutes (range: 10–39 minutes). A semistructured focus group guide was designed by the research team to facilitate the focus group discussions. The questions in the focus group guide were divided into three topics to collect data for two separate studies. The first two topics were related to how adolescents navigated their food environment, of which the results are reported in another study by Mesch *et al*. (2025b). For the current study, only questions about the third topic were used for analysis, exploring how important students perceive healthy and sustainable food. The questions regarding all topics are shown in Table 1.

**Table 1:** Focus group guide topic and questions.

Topic	Questions
First impression and comparison	Compare your assignment with those of others in the group. Which differences do you see? Why do you think there are differences?
Food outlets: frequency and influences	Which outlets did many of you indicate? Why do you obtain food from these places? Do you take food with you to school from home? Why or why not? Why did you give some places a lot of stars? Or only a few stars? Are you satisfied with the food you can get at school, around school, and at home? Why or why not?
Healthy and sustainable food ‘(this study)’	At which places is healthy and sustainable food available and at which places not? At which places do you eat healthy and sustainable food and at which places not?
	How important is healthy and sustainable food for you? Why is it important, or why is it not important?
	Do you consider health and/or sustainability when you make food choices? If so, do healthy food and sustainable food always go together? If health and sustainability contradict, how do you make a choice?

#### Classroom discussions: understanding of healthy and sustainable food

The following session in class started with an exploration of the concepts of healthy and sustainable food. Firstly, researcher A.M. asked students for their associations with the concepts of healthy food and sustainable food. Secondly, students were asked whether they knew any arguments for eating healthy food and sustainable food. Students contributed in class on a voluntary basis. Researcher F.H. made field notes of the answers provided by students in the classroom. The sessions were not audio-recorded. After students responded to the questions, A.M. provided additional information on healthy and sustainable food [based on recommendations by the Dutch Nutrition Centre (Voedingscentrum)] to ensure that students had similar baseline knowledge to continue with the next assignment.

#### Group assignment: proposed strategies to facilitate healthy and sustainable diets

Students were divided into 106 groups of two to three students to work on an assignment in which they were prompted to develop a strategy that facilitates healthy and sustainable eating in their age group. The instructions stimulated students to think about solutions on different levels of the socioecological model (Story *et al.*, 2008): on the individual level (i.e. ‘what could you change?’), in the social environment (i.e. ‘what could others, e.g. caregivers or peers, change?’), in the physical environment (i.e. ‘what could be changed in the food environment?’), and in the macrolevel environment (i.e. ‘what could be changed on a structural level, e.g. in politics or marketing?’). Groups were asked to first fill out a worksheet that helped them specify their idea. This worksheet started with the main question ‘What can help you to eat (more) healthy and sustainable food?’, followed by six subquestions based on the ‘5W1H’ method including questions related to the ‘why’, ‘who’, ‘where’, ‘when’, and ‘how’ (Supplementary Table S3). They were free to propose any kind of solution, whether focused on a single level of the socioecological model or on multiple levels.

After filling out the worksheet, all groups made a poster illustrating their proposed strategy. Following this, students pitched their strategies (∼1–2 minutes) to at least one other group of students and one of the researchers or research assistants. These pitches were audio-recorded.

### Data analysis

In this mixed-methods study, quantitative and qualitative data were gathered simultaneously. Figure 1 shows how the diverse methods used in this mixed-methods study were integrated and contributed to the different aspects of our research question. To explore adolescents’ ‘understanding’ of healthy and sustainable food, classroom discussions were analysed to explore themes that adolescents associate with the concepts of healthy and sustainable food. Moreover, this data from classroom discussions provided themes regarding reasons why one would eat healthy and sustainable food. The quantitative data were analysed to understand the ‘perceived importance’ of healthy and sustainable food, which was further illustrated by the qualitative data from the focus groups. Additionally, to explore ‘proposed intervention strategies’ to facilitate healthy and sustainable diets, qualitative data from the worksheets, posters, and pitches were analysed.

#### Quantitative analysis of questionnaire data: perceived importance of healthy and sustainable food

Demographic characteristics resulting from the questionnaires were evaluated through descriptive analyses, to describe the study sample. Dietary restrictions (e.g. vegetarian, intolerances, and halal) were grouped into two categories: ‘yes’ and ‘no’. Differences in demographic characteristics between urban and rural areas were tested using a Mann–Whitney *U* test (age), chi-square tests (educational track, dietary restrictions, and money expenditure), and Fisher’s exact tests (gender). Differences in the perceived importance of healthy and sustainable food between rural and urban areas were explored using ordinal logistic regressions while correcting for educational track, dietary restrictions, and money expenditure. All analyses were performed using statistical software R version 4.4.0.

#### Qualitative analysis of focus groups: perceived importance of healthy and sustainable food

After intelligent verbatim transcription, focus group data was analysed using ATLAS.ti version 22. Coding took place in multiple rounds. Firstly, researchers A.M. and F.H. independently coded two transcripts through inductive coding. After coding individually, A.M. and F.H. compared coding schemes and reached consensus on an initial shared coding scheme. Following this, A.M. coded another 18 transcripts using and expanding the coding scheme. A renewed version of the coding scheme was then discussed with F.H. and adapted. Another 11 transcripts were then coded by A.M. and discussed afterwards with F.H. As no additional main codes came up in the last six transcripts, the researchers concluded that data saturation was reached. F.H. coded one final transcript to check and approve the coding scheme. All analysed transcripts (*n*  *=* 32) were from researcher-led focus groups and represented different classes of all four schools. The remaining researcher-led focus groups (*n* = 10), as well as the peer-led (*n* = 7) and teacher-led (*n* = 1) focus groups, were not coded anymore, but were listened to by A.M. to check if no themes were missed. Supplementary Figure S4 provides an overview of the coding process.

#### Qualitative analyses of classroom discussions: understanding of healthy and sustainable food

The field notes that were made during the research were evaluated by researcher A.M., in order to inductively code and categorize the answers of students relating to their understanding on what healthy and sustainable food entails and on reasons to eat healthy and sustainable food. This categorization was checked by F.H., and any disagreements were discussed and resolved.

#### Qualitative analysis of group poster assignment: proposed strategies to support healthy and sustainable food choices

After intelligent verbatim transcription of the pitches of two schools, the worksheets, posters, and transcripts were analysed together to gain insight into what codes could be developed from the ideas of students. Researchers A.M. and F.H. together assessed the data of two schools and coded the data inductively after the first round of data collection in spring 2023. These codes were then grouped into 13 themes. The data of the other two schools were analysed after the second round of data collection in autumn 2023 by researcher A.M. using the initial coding schemes while listening to the pitches and analysing the worksheets and posters. If new themes occurred, they were added to the coding scheme and were discussed with F.H. until consensus was reached. Finally, in consultation with the research team, we divided the 13 themes into four categories to present the results. Two posters were excluded because they were considered out of the scope of the assignment.

Additionally, the rationales for the proposed strategies on the posters, which were formulated on the worksheets as an answer to the question ‘why do you want to change this?’ were analysed deductively by A.M., using the dimensions of the FAO definition of a healthy and sustainable diet. The answers were then coded as ‘health dimension’, ‘environmental dimension’, or ‘sociocultural dimension’. Those categories were not mutually exclusive; multiple dimensions could be coded in one answer. The answers that did not fit under any of these codes were coded inductively. F.H. checked the coding, and any disagreements were discussed and resolved.

### Reflexivity statement

The authors work together in an interdisciplinary team, with expertise in public health nutrition (A.M., F.H., and A.H.-N.), social psychology (S.R.), behavioural sciences (L.H.H.W. and S.R.), education sciences (J.G. and R.W.), and epidemiology (A.H.-N.). This interdisciplinary experience and composition of this team supported the multidimensionality of this research topic, given that together the team has expertise on healthy and sustainable food, behaviour change, learning, and teaching. This interdisciplinary expertise provided useful insights for the study design, development of materials fitting the secondary school setting, and analysis of the different datasets. The team also has a strong link with practice, given that F.H. works at a practice-based research institute, A.H.-N. has a position at the municipality health services, and J.G. is involved in teacher training. Although none of the researchers or research assistants who collected the data were trained teachers, most of them had some experience in the school setting or working with youth.

## Results

### Study population

Table 2 presents an overview of participants’ demographic characteristics. The average age of participants was 13.5 years (range: 12–16 years old). There was a slightly higher proportion of females than males and a higher proportion of students participating from ‘senior general’ classes than ‘prevocational’ or ‘preuniversity’ classes (Table 2). In rural areas, more students from ‘prevocational’ (41%) and ‘senior general’ classes (40%) participated than from ‘preuniversity’ classes (20%). In urban areas, most students participated from ‘senior general’ classes (41%), compared to ‘preuniversity’ (34%) and ‘prevocational’ classes (25%). Overall, the majority of students (68%) indicated that they spend their own money on food at least once a week. Of those students who spend money on food weekly, two-thirds indicated to do so at least two times per week. More than a fifth of the entire sample (23%) indicated having some special dietary restrictions. Of all dietary restrictions reported, following a halal diet was most often reported (40%).

**Table 2:** Demographics of adolescents in study population (*n* = 296).

	Total (*n* = 296)	Rural (*n* = 133)	Urban (*n* = 163)
Age, mean ± SD	13.5 ± 0.9	13.6 ± 1.1	13.5 ± 0.8
Gender, *n* (%)			
Female	158 (53)	70 (53)	88 (54)
Male	129 (44)	60 (45)	69 (42)
Nonbinary	2 (1)	2 (2)	0 (0)
Other	7 (2)	1 (1)	6 (4)
Educational track, *n* (%)^a^**			
Prevocational secondary education (vmbo)	95 (32)	54 (41)	41 (25)
Senior general secondary education (havo)	120 (41)	53 (40)	67 (41)
Preuniversity education (vwo)	81 (27)	26 (20)	55 (34)
Dietary restrictions, *n* (%)***			
Yes	68 (23)	17 (13)	51 (31)
No	226 (76)	115 (86)	111 (68)
Missing	2	1	1
Money expenditure on food, *n* (%)**			
Once per month or less	47 (16)	25 (19)	22 (13)
Once per 2 weeks	38 (13)	25 (19)	13 (8)
Once per week	75 (25)	35 (26)	40 (25)
Two to four times per week	102 (34)	34 (26)	68 (42)
Every school day	28 (9)	9 (7)	19 (12)
Missing	6	5	1

^a^Educational track refers to the Dutch educational system, including vmbo (‘prevocational secondary education’), havo (‘senior general secondary education’), and vwo (‘preuniversity education’).

The significant differences between the rural and urban groups are reported on the following levels: **P < 0.01, and ***P < 0.001

### Adolescents’ understanding and perceived importance of healthy and sustainable food

#### Understanding of healthy and sustainable food

**Figure 2:**
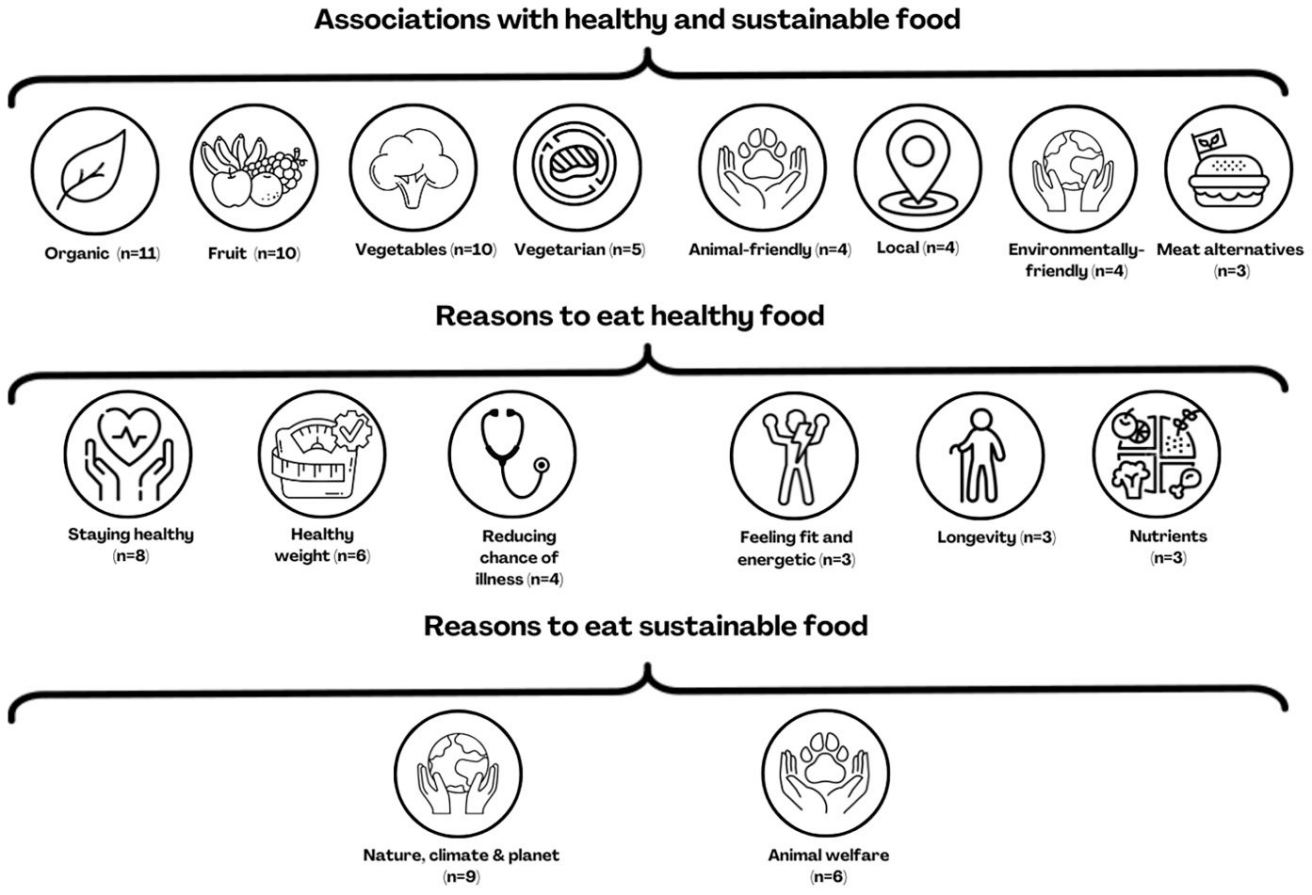
Overview of most mentioned associations (≥3) with healthy and sustainable food, reasons to eat healthy food, and reasons to eat sustainable food (*n* = 13 classes).

Figure 2 shows themes that students mentioned most (in at least three classes) during in-class discussions regarding healthy and sustainable food and underlying reasons. The most mentioned themes regarding healthy and sustainable food were organic food (in *n* = 11 out of 13 classes), fruit (*n*  *=* 10), and vegetables (*n*  *=* 10). Other themes, such as vegetarian food (*n*  *=* 5) and food that is produced with consideration for animal welfare (*n*  *=* 4), were also mentioned. When asked for reasons why one would eat healthy food, students primarily mentioned staying healthy (*n*  *=* 8), maintaining a healthy weight (*n*  *=* 6), and reducing chance of illness (*n*  *=* 4*).* Regarding the reasons why one would eat sustainable food, protecting nature, climate, and planet (*n*  *=* 9), and animal welfare (*n*  *=* 6) were mentioned.

#### Perceived importance of healthy and sustainable food

Following from the questionnaire that students individually filled out, two-thirds of the sample (68%) perceived it (very) important to eat healthy food, whereas a fifth (21%) indicated that they find it (very) important to eat sustainable food (Table 3). More than one-tenth (12%) of the sample indicated not knowing what sustainable food entails. There were significant differences regarding the importance of healthy and sustainable food between groups, corrected for educational track, dietary restrictions, and money expenditure on food. Being in the urban group was associated significantly with higher odds of perceiving healthy food as more important than being in the rural group [Odds Ratio (OR) = 2.34; 95% Confidence Interval (CI) (1.43, 3.86); *P* = 0.001], but nonsignificantly with higher odds of perceiving sustainable food as more important [OR = 1.58; 95% CI (0.96, 2.62); *P* = 0.073]. Moreover, an increase in frequency of money expenditure was associated with a lower odds of perceiving healthy food as important [OR = 0.12; 95% CI (0.06, 0.26); *P* < 0.001] and with a lower odds of perceiving sustainable food as important [OR = 0.43; 95% CI (0.19, 0.95); *P* = 0.038]. Moreover, being in a ‘senior general’ class was associated with higher odds of perceiving sustainable food as important than being in a ‘prevocational’ class [OR = 1.89; 95% CI (1.07, 3.35); *P* = 0.028]. Being in a ‘preuniversity’ class was also associated with a higher odds of perceived sustainable food as important than being in a ‘prevocational’ class [OR = 3.13; 95% CI (1.66, 5.97); *P*  *<* 0.001].

**Table 3:** Adolescents’ perceived importance of healthy and sustainable food (*n* = 296)^a^.

	Total (*n* = 296)	Rural (*n* = 133)	Urban (*n* = 163)
Importance of healthy food, *n* (%)***			
Not important at all	6 (2)	3 (2)	3 (2)
Not so important	22 (8)	12 (9)	10 (6)
Neutral	65 (22)	40 (30)	25 (16)
Important	159 (54)	61 (46)	98 (61)
Very important	39 (13)	16 (12)	23 (14)
I don’t know what healthy food is^b^	2 (1)	0 (0)	2 (1)
Missing	3	1	2
Importance of sustainable food, *n* (%)			
Not important at all	35 (12)	16 (12)	19 (12)
Not so important	61 (21)	39 (30)	22 (14)
Neutral	102 (35)	47 (36)	55 (34)
Important	49 (17)	13 (10)	36 (22)
Very important	11 (4)	5 (4)	6 (4)
I don’t know what sustainable food is^b^	35 (12)	12 (9)	23 (14)
Missing	3	1	2

^a^Analyses are adjusted for educational track, money expenditure, and dietary restrictions.

^b^This category was not taken into account for analysis, as it does not reflect perceived importance.

The significant differences between the rural and urban groups are reported on the following level: ***P < 0.001

Along the same lines, students responded more positively to the importance of healthy food than to the importance of sustainable food during the focus group discussions. Nevertheless, students described that when they themselves choose food outside of their homes, they mostly do not consider its healthiness:

It depends, if I just go to the supermarket, then I just buy a tasty sandwich. Then I don’t really care [how healthy it is].

—Student school 2 (rural, preuniversity class)

Moreover, for both healthy and sustainable food, students perceived their caregivers to have the main responsibility. To illustrate, students mentioned that they think their parents ensure that they eat healthy and/or sustainable food at home or follow their caregivers’ ideas about health and sustainability:

I: Do you think about healthy food at home or do you think “there I will get healthy food anyway”?

P: I hope my Mum thinks about it.

—Student school 1 (rural, prevocational class)

P: In my culture sustainable food is normal. My Mum makes her own bread. I think it is also…

I: That you make more by yourselves and you buy less?

P: Yes. It is also fresh and healthier I think.

—Student school 4 (urban, prevocational class)

Nevertheless, students mentioned several (personal) reasons why they would choose healthy food themselves, including not wanting to have an extremely unhealthy dietary pattern, not wanting to eat unhealthy food for breakfast or during sports, and preventing weight gain or the development of illnesses. Interestingly, regarding sustainable food, students explained that they felt like eating sustainable food is beyond their control and that they are lacking the knowledge to actually do so:

P: I don’t think about sustainability, because it is not because of me that the food is not sustainable. It’s because of other people.

—Student school 4 (urban, prevocational class)

I: You’re saying that you do not eat sustainably. Do you know why not?

P: Yes, because we do not know so much about it

—Student school 4 (urban, prevocational class).

### Adolescents’ proposed strategies facilitating healthy and sustainable food choices

Through 104 posters and pitches, students expressed their ideas for strategies facilitating healthy and sustainable food choices among their age group. Themes derived from those ideas are discussed below according to four categories: ‘strategies to change the food environment, strategies to change the food system, strategies for communication and social support, and individual behaviour change strategies’.

Before visualizing their proposed strategies on posters, students formulated the rationale behind their strategies on their worksheets. These rationales included health dimensions (i.e. individual wellbeing), environmental dimensions (i.e. environmental pressures), and sociocultural dimensions (i.e. affordability, equitability, and acceptability), as conceptualized in the definition of healthy and sustainable diets by the FAO and WHO (2019) (Food and Agriculture Organisation and World Health Organisation 2019). To illustrate, regarding the health dimension (*n* = 44), students argued that they want to promote healthy food so that people stay healthy or feel fit and energetic. Regarding the environmental dimension (*n* = 31), students emphasized that it is important to save the planet or to prevent further environmental degradation. Regarding sociocultural dimensions (*n* = 44), students mainly emphasized the importance of a fair price for the producers and that healthy and/or sustainable food should be affordable for all:

So that people with little money can also afford healthy food.

—Group at school 3 (urban, senior general class)

Additionally, seven groups mentioned improving animal welfare (*n*  *=*  *7*) as a rationale for their proposed strategies.

#### Proposed strategies to change the food environment

**Figure 3:**
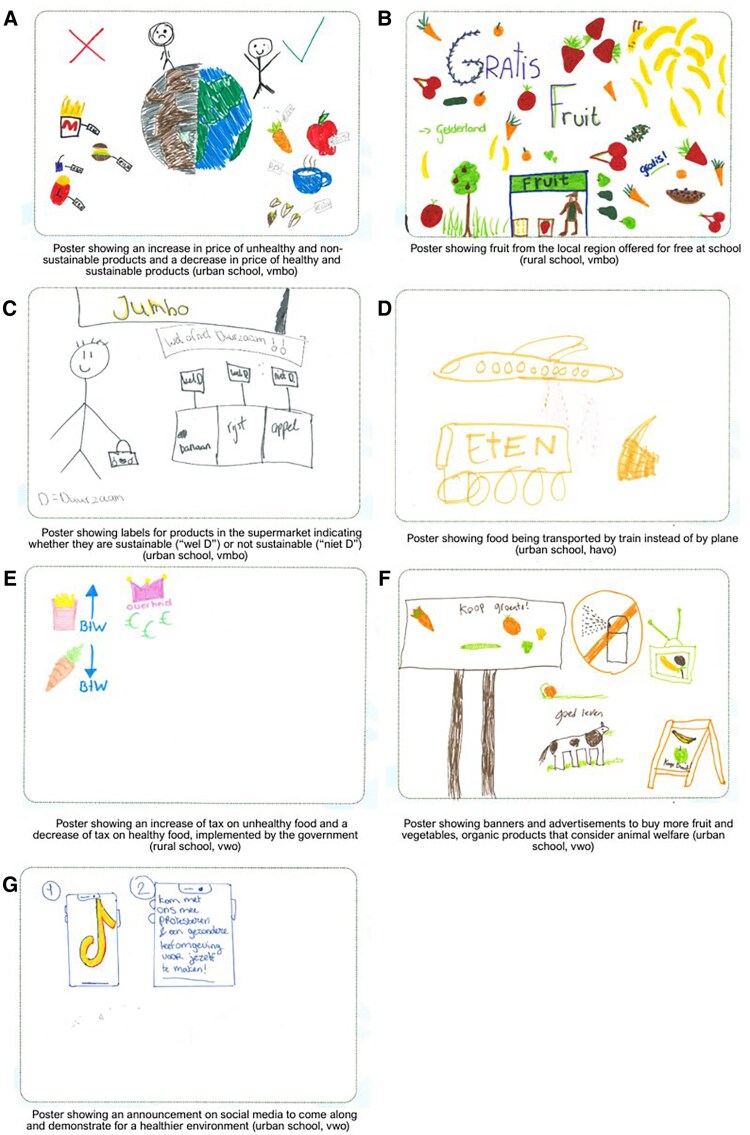
Posters (A-G) illustrating adolescents’ ideas for strategies facilitating healthy and sustainable food choices among their age group.

Regarding strategies to change ‘the food environment’, most groups suggested strategies that were related to the ‘price of food (*n*  *=* 74)’. Students suggested decreasing the price of healthy and sustainable food and/or increasing the price of food that is unhealthy and not sustainable (Fig. 3a). Proposed examples of ways to change the price of food were discounts in supermarkets for healthy products and offering free food at schools.

Besides, students suggested to ‘increase the offer of healthy and sustainable food (*n*  *=* 30)’. For example, they proposed healthy and sustainable alternatives to current food outlets (e.g. healthy restaurants instead of fast food) as well as more healthy and sustainable options in various food outlets, including the supermarket or the school canteen (Fig. 3b). Students also suggested a different ‘positioning and presentation of food (*n* = 6)’, e.g. by changing the choice-architecture (i.e. placing healthy or sustainable products in front), developing new labels for sustainable or organic products to improve recognition of those products (Fig. 3c), and reducing portion sizes. Taken together, these suggestions show that adolescents recognize multiple leverage points in their food environment, related to financial, offer-related, and presentational aspects to promote healthier and more sustainable food choices.

#### Proposed strategies to change the food system

Students proposed strategies related to upstream drivers such as production, processing, transport, and regulations, which we refer to as the ‘food system’. Regarding the ‘food system’, students suggested ‘more sustainable food production (*n*  *=* 23)’, such as increasing local food production (e.g. only regional/national products), increasing sustainable/organic agriculture (e.g. no use of pesticides), and stimulating people to produce their own food (e.g. in vegetable gardens at school). Furthermore, students thought of strategies to ‘improve animal welfare (*n*  *=* 4)’ by ensuring better and longer living conditions for animals. They also proposed strategies to ensure ‘more sustainable transport (*n*  *=* 6)’, such as transporting food by train or electric vehicles (Fig. 3d). Besides, students proposed strategies related to ‘alternative packaging (*n*  *=* 8)’, such as reusable packaging or using alternatives to plastic packaging. Regarding the broader system, students argued that the ‘government should implement regulations facilitating healthy and sustainable food options (*n*  *=* 20)’, such as reducing taxes on organic products and increasing taxes on nonorganic products (Fig. 3e), investing in healthy and sustainable alternatives to fast food, and providing subsidies to producers of healthy food and organic food. Taken together, these suggestions show that adolescents recognize multiple possible actions in their food system, related to production, transport, packaging, and governmental action to facilitate the production of healthier and more sustainable food.

#### Proposed strategies for communication and social support

Regarding strategies for ‘communication and social support’, students mentioned strategies related to ‘advertisements for healthy and sustainable food choices (*n*  *=* 10)’, such as advertisements on television or banners for organic products, vegetables, or products with animal welfare certifications (Fig. 3f). Additionally, students mentioned ‘a supportive social environment (*n*  *=* 3)’, such as having people in their social environment who set the right example. Moreover, students mentioned ‘integrating the theme in their schools (*n*  *=* 6)’, either through education from their teachers or through challenges among their classmates. For example, students proposed a challenge to buy the healthiest food product(s) with a certain budget or to eat the highest amount of fruit and the lowest amount of meat during a certain period. Lastly, students mentioned the importance of ‘creating societal support for change (*n*  *=* 10)’. To illustrate, students suggested organizing petitions to gather signatures for a desired change (e.g. lowering the price of strawberries) or posting announcements on social media to go demonstrate for a desired change (e.g. a healthier environment; Fig. 3g). Taken together, these suggestions show that adolescents recognize opportunities to better support healthy and sustainable food choices, through advertisements, supportive social environments, and creating societal support.

#### Proposed strategies for individual behaviour change

Finally, six groups proposed that they should ‘individually change their dietary behaviour (*n*  *=* 6)’, such as by avoiding unhealthy foods, making grocery lists with healthy products, and balancing unhealthy and healthy foods.

#### Counterperspectives

Three groups showed a counterperspective on the poster assignment, arguing that they did not want to propose a solution to facilitate healthy and sustainable eating. They explained that they wanted more meat production, that they thought meat alternatives (based on soy) have a bad impact on the environment, and that they thought everyone should have their own opinion and autonomy regarding what they eat.

#### Variation in ideas across geographic location and educational track

Overall, all themes that were mentioned by many groups came forward in posters at schools in urban and rural areas and across all educational tracks. The ‘government implementing regulations to facilitate healthy and sustainable food’ (*n* = 20), mentioned under strategies to facilitate the food system was, apart from one class, primarily found at urban schools. Some themes that were mentioned by six or fewer groups were found only in one region or in one educational track. Given this low number, we do not mention those differences here.

## Discussion

This mixed-methods study explored adolescents’ perspectives on healthy and sustainable food, in terms of understanding, perceived importance, and proposed strategies to facilitate healthy and sustainable dietary behaviours. Our findings indicate that students primarily understood healthy and sustainable food as organic food, vegetables, and fruits. The most frequently mentioned arguments for eating healthily and sustainably included maintaining good health, maintaining a healthy weight, protecting the climate, and improving animal welfare. While two-thirds of the sample found eating healthy food (very) important, only one-fifth attached the same level of importance to sustainable food. Moreover, more than a tenth of the sample indicated not knowing what sustainable food entails. Overall, adolescents proposed strategies for facilitating healthy and sustainable food related to four categories: ‘strategies to change the food environment, strategies to change the food system, strategies for communication and social support, and strategies for individual behaviour change’. Strategies to change the food environment, including a change in price and a change in the offer of food, and strategies to change the food system, including more sustainable food production and governmental regulations, came forward most. Those strategies were mostly proposed based on health-related and sociocultural arguments and to a slightly lesser extent on environmental arguments.

Consistent with Gisslevik *et al*. (2019), adolescents formulated healthy and sustainable food as tangible and close-to-home terms—i.e. specific food groups such as fruits, vegetables, and organic products—rather than the broader systemic and abstract aspects of sustainability (Gisslevik *et al*. 2019). Given that we asked students for their understanding of healthy and sustainable food rather than the broader food system, it may be evident that students are more likely to refer to more concrete food products. Besides, following from the data from in-class discussions, adolescents were better able to directly articulate their views on healthy food than sustainable food in class, which is consistent with earlier findings (Teixeira *et al*. 2024, Lanham and Van der Pols 2025). This discrepancy between students’ relation with health aspects and sustainability aspects of food is further reflected in the higher scores on perceived importance of healthy food than of sustainable food, the wider variety of reasons adolescents reported for eating healthy food than for sustainable food, and the higher number of poster assignments that focused on improving health. Nevertheless, students demonstrated a more thorough understanding of sustainable food in the poster assignments than in classroom discussions, touching upon wider food system aspects, such as transport, food production (e.g. organic), animal welfare, packaging, and equity considerations. This shows that even though it may be challenging for adolescents to concretely describe sustainable food, they are able to come up with concrete solutions. As such, adolescents’ understanding of sustainable food may be better developed than previously suggested in prior research (Lanham and Van der Pols 2025).

Additionally, prior research indicated that adolescents tend to address health and ecological dimensions when defining healthy and sustainable diets, but do not address economic and social dimensions such as those mentioned in the definition of the FAO and WHO (Teixeira *et al*. 2024, Lanham and Van der Pols 2025). In the current study, sociocultural dimensions (i.e. affordability, equitability, and acceptability) were not mentioned in any description of sustainable food but were addressed in many poster rationales. For example, students referred to equal accessibility, fair pricing, and animal welfare as arguments for their proposed intervention strategies. This suggests that, in addition to health and environmental considerations, sociocultural and ethical concerns, such as animal welfare or justice, may hold potential to engage and motivate adolescents to work on more healthy and sustainable diets. Given that this age group appears to be particularly engaged with issues of justice and social responsibility (Fuligni 2019, Thomaes *et al*. 2023), future studies could explore whether emphasizing sociocultural and ethical dimensions may provide a viable (alternative) entry point for stimulating healthy and sustainable diets among adolescents.

In the current study, the strategies that adolescents proposed to promote healthy and sustainable diets primarily addressed the offer and price of food. This focus may not be surprising, as price and food offer are tangible facets of the food environment and therefore may easily come to mind (Li and Swinburn 2025). Moreover, those proposed strategies align with prior research showing that the costs and availability of food play a crucial role in how adolescents navigate their food environment (Kelly *et al*. 2021, Neufeld *et al*. 2022, Ryan *et al*. 2022, Raghoebar *et al*. 2024, Mesch *et al*. 2025b). For example, changing food offer and price also came forward in previous studies exploring adolescents’ ideas for stimulating healthy eating, studied in Europe and Latin America (Ares *et al*. 2021, Conway-Moore *et al*. 2023). However, in those studies, most strategies proposed by adolescents were related to communication and education, such as raising awareness through social media campaigns or increasing nutrition education for students and families. While some posters in the current study mentioned communication strategies (i.e. advertisements and nutrition education at school), those were less frequently proposed than more structural strategies targeting the food environment. This may be explained by a difference in the local contexts of this study compared to other studies. To illustrate, in the Dutch school context, there are no universal school meals and students have more agency to make own food choices, which may explain why students focus on solutions regarding food offer and prices that directly influence their daily food choices. All in all, our findings clearly illustrate the desire of adolescents in the Dutch secondary school context to be provided with a more healthy and sustainable food offer that is also affordable.

Interestingly, even though autonomy and social processes (e.g. social status) are generally regarded as important values during adolescence and are key drivers of adolescent food choices (Kelly *et al*. 2021, Neufeld *et al*. 2022, Ryan *et al*. 2022, Raghoebar *et al*. 2024), those factors were not clearly reflected in adolescents’ proposed strategies. According to the motivation-alignment hypothesis by Thomaes *et al*. (2023), interventions promoting sustainable behaviour are more likely to be successful when they align with autonomy and social status (Thomaes *et al*. 2023). Similarly, the self-determination theory (SDT) states that motivation is enhanced when the needs for autonomy, relatedness, and competence are met (Ryan and Deci 2000). Yet, in this study, adolescents did not strongly perceive healthy and sustainable food choices as their own responsibility. To illustrate, adolescents often referred to the role of caregivers, and adolescents who spent money on food more often had a lower odds of perceiving healthy and sustainable food as important. Moreover, most proposed strategies involved changing the system or the food environment, which require actions of others, such as the government, the school, or food retailers, rather than themselves. Only a small number of groups proposed strategies involving an active role for themselves, either through individual behaviour change or through mobilizing the community or peers to take action. Nevertheless, previous research on youth-led advocacy to change aspects of the food environment, such as a youth-led cafeteria food labelling and social marketing campaign, have shown promising results, including increased knowledge on healthy foods and increased fruit and vegetable intake (Frerichs *et al*. 2015). Regarding social aspects, only a couple of groups proposed strategies including social elements, such as strengthening social support or introducing peer-based food challenges. Nevertheless, none of the groups proposed solutions that were related to mealtime ambiance, having a social lunch time with peers, or changing norms, which are factors that adolescents illustrated as important in previous studies (Ryan *et al*. 2022, Raghoebar *et al*. 2024, Mesch *et al*. 2025b).

The absence of autonomy and social processes in adolescents’ proposed strategies may be explained by several factors. First of all, autonomy and social processes are less concrete factors than factors in the physical environment (e.g. offer and price), making it more complicated for adolescents to identify and translate into concrete strategies (Li and Swinburn 2025). Moreover, not perceiving full personal responsibility is in line with the development of autonomy during adolescence, a period in which adolescents experience various degrees of autonomy and responsibility for their own (dietary) choices (Ziegler *et al*. 2021). Adolescents are known for questioning imposed demands, which could also motivate them to make unsustainable and unhealthy choices (Raghoebar *et al*. 2024). This was especially visible in a few posters that showed counterarguments, such as not wanting to see any changes to stimulate more healthy and sustainable food choices. Additionally, in the current study, adolescents indicated having insufficient knowledge about sustainable food, and perceiving sustainability issues as beyond their control, indicating low feelings of competence to make sustainable food choices. This is in line with the SDT as stated above, where competence is one of the essential factors for enhancing motivation, alongside autonomy and relatedness (Ryan and Deci 2000, Miketinas *et al*. 2016). Hence, efforts are needed to make sustainable food more personally relevant for students and show in which way they could contribute to a more sustainable food system.

The abovementioned findings emphasize the importance of engaging adolescents in the development of interventions and making the topic applicable to adolescents’ daily lives instead of a distant (future-oriented) topic. Active participation may reinforce their feeling of autonomy and competence and thereby empower them to be part of future solutions (Cebrián *et al*. 2025). Moreover, our current findings show that adolescents are well able to think about solutions to promote healthy and sustainable food choices. At the same time, while engaging adolescents is essential, it is also important to avoid a mere focus on individual responsibility (Carey *et al*. 2017, Raghoebar *et al*. 2024). Overemphasizing personal responsibility may result in adolescents feeling unsupported and may reinforce the perception that complex solutions are beyond their control (Stevenson and Peterson 2016). Hence, it seems most promising to cocreate strategies facilitating healthy and sustainable food choices with adolescents and stakeholders (e.g. teachers, food retailers, and policy makers) to create a feeling of shared responsibility and to ensure that strategies match adolescents’ values and lived experiences. Moreover, as in the current base of literature there is no consensus on the most successful way of promoting healthy and sustainable diets, cocreation can help develop solutions that build on adolescents’ understanding of the topic and are tailored to the diverse contexts in which they navigate.

### Strengths and limitations

This study uniquely contributed to our understanding of adolescents’ own perspectives on ways to facilitate healthy and sustainable food choices, which has (until now) often not been taken into account when developing food interventions for this age group. A key strength of this study is the combination of multiple methods, including visual methods (i.e. posters), verbal methods (e.g. classroom discussions, focus groups, and pitching), and written methods (i.e. worksheet and questionnaire). Using this combination of methods made the study more inclusive by facilitating multiple communication styles and strengthened the findings through cross-validation.

Nevertheless, data on adolescents’ understanding of healthy and sustainable food was mainly gathered from classroom discussions, where group dynamics played a role. In some classes, students were more hesitant to speak up and actively participate in those classroom discussions, which may have limited our exploration of students’ understanding of healthy and sustainable food. Future studies may benefit from further exploring tailored methods that better support students in formulating their perspectives, given that students’ understanding of healthy and sustainable food was not captured well in classroom discussions. Instead, working on a concrete assignment such as the poster assignments in this study, instead of answering a broad question in class, may be a better approach to capture their understanding.

We purposely recruited a large and diverse sample at four different secondary schools in urban and rural areas in the Netherlands, including students of all educational tracks of the regular Dutch secondary school system. The difference in themes found in the posters between the geographical locations may be related to the different times of data collection (spring versus autumn 2023). In autumn 2023, Dutch elections were held, which may have drawn more emphasis on the role of governmental actions. Nevertheless, we were unable to explore the diversity of our sample regarding other aspects that are known to relate to dietary choices, such as culture and socioeconomic position, as collecting such data from minors was inappropriate for the scope of this research. For future interventions to be inclusive and equitable, it is essential to explore how those can be responsive to the needs of students from various cultural and socioeconomic backgrounds.

## Conclusion

In this mixed-methods study, healthy food was better understood and perceived as more personally relevant than sustainable food by adolescents. To increase the relevance of sustainable food, connecting to sociocultural and ethical values they care about, such as equity, may hold particular promise. In terms of future strategies, adolescents emphasized changing the food environment and the food system, with a clear call for actions by governments and industries to ensure a healthy, sustainable, and affordable food offer. Given those ideas, our findings underscore the importance of cocreating future strategies together with adolescents and other stakeholders on various levels of influence, among which are policymakers, food retailers, and school staff. Such an approach has the potential to design strategies that address broader structural factors while remaining responsive to the diverse contexts and lived experiences of adolescents.

## Supplementary Material

daaf230_Supplementary_Data

## Data Availability

The datasets analysed during the current study are not publicly available as this would violate participant consent. The analysis plans and coding schemes are available from the corresponding author on reasonable request.
