# Supplementary material for: Making sense of healthy and sustainable food: adolescents’ voices on what it means, why it matters, and future change
Source: Health Promot Int. 2026 Jan 31;41(1):daaf230. doi: 10.1093/heapro/daaf230 (PMC12858370; doi:10.1093/heapro/daaf230)
Supplement: daaf230_Supplementary_Data [file daaf230_supplementary_data.docx]

**Supplementary Materials**

**Table S1.** Characteristics of participating schools (*n=4*)

| **School** | **Area** | **Educational track^a^** | **No. of participating classes** | **Grade** | **Time of data collection** | **School food policy** | **Certification (e.g. the Dutch ‘Healthy School’ certificate)** | **Attention for food in school/curriculum** |
| --- | --- | --- | --- | --- | --- | --- | --- | --- |
| School 1 | Rural | vmbo | 2 | 3 | Spring 2023 | No | No | Yes, in home economics classes |
| School 2 | Rural | vmbo, havo, and vwo | 4 | 1-3 | Spring 2023 | Healthy canteen policy, restriction to leave school area for first grade | No | Yes, in coaching hours |
| School 3 | Urban | havo and vwo | 5 | 2 | Autumn 2023 | Healthy canteen policy, restriction to leave school area for first grade | No | Yes, in biology classes |
| School 4 | Urban | vmbo | 2 | 3 | Autumn 2023 | Healthy canteen policy, restriction to leave school area for all grades | No | Yes, in care and welfare classes |
| ^a^ Educational track refers to the Dutch educational system, including vmbo (*pre-vocational secondary education*), havo (*senior general secondary education*) and vwo (*pre university education*) | | | | | | | | |

**Supplementary Materials S2. English version of the questionnaire for adolescents participating in this study**

| Class | | |  |
| --- | --- | --- | --- |
| Age (in years) | | |  |
| How do you identify yourself? I identify myself as: | | | |
| O | Female | | |
| O | Male | | |
| O | Non-binary | | |
| O | Other | | |
| Do you follow any dietary prescriptions? Select the option(s) that apply to you: | | | |
| O | Vegetarian (I **do not** **eat** meat and fish; but I **do** **eat** milk and eggs) | | |
| O | Pescetarian (I **do not eat** meat; but I **do eat** milk, eggs and fish) | | |
| O | Vegan (I **do not eat** meat, fish, milk and eggs) | | |
| O | Halal | | |
| O | Kosher | | |
| O | Gluten-free | | |
| O | Lactose-free | | |
| O | Another allergy or intolerance, namely: .………………………………………….. | | |
| O | Another dietary prescription, namely: …………………………………………….. | | |
| O | None of these options | | |
| How often do you spend your own money on food, snacks and beverages (for example your pocked money or money you earn with a side job)? For example, think about what you buy in school breaks, a free hour or after schooltime? | | | |
| O | (Almost) never | | |
| O | Once a month | | |
| O | Once every two weeks | | |
| O | Once per week | | |
| O | Two up to four times a week | | |
| O | Every school day | | |
| How important is it for you to eat healthy food? | | | |
| O | Not important at all | | |
| O | Not very important | | |
| O | Not important/not unimportant | | |
| O | Important | | |
| O | Very important | | |
| O | I do not know what *healthy food* entails | | |
| How important is it for you to eat *sustainable food*? | | | |
| O | Not important at all | | |
| O | Not very important | | |
| O | Not important/not unimportant | | |
| O | Important | | |
| O | Very important | | |
| O | I do not know what *sustainable food* entails | | |

**Table S3.** Questions on the worksheet to be answered by students

| **Kipling’s principle (5W1H)** | **Question on worksheet** |
| --- | --- |
| ***What?*** | What do you want to change? |
| ***Why?*** | Why do you want to change this? |
| ***How?*** | How do you want to bring about this change? |
| ***Who?*** | Who will you need to bring about this change? |
| ***Where?*** | Is this idea bound to one specific food outlet (e.g. home, supermarket)? |
| ***When?*** | Is this idea bound to a specific meal (e.g. lunch, snacks)? |


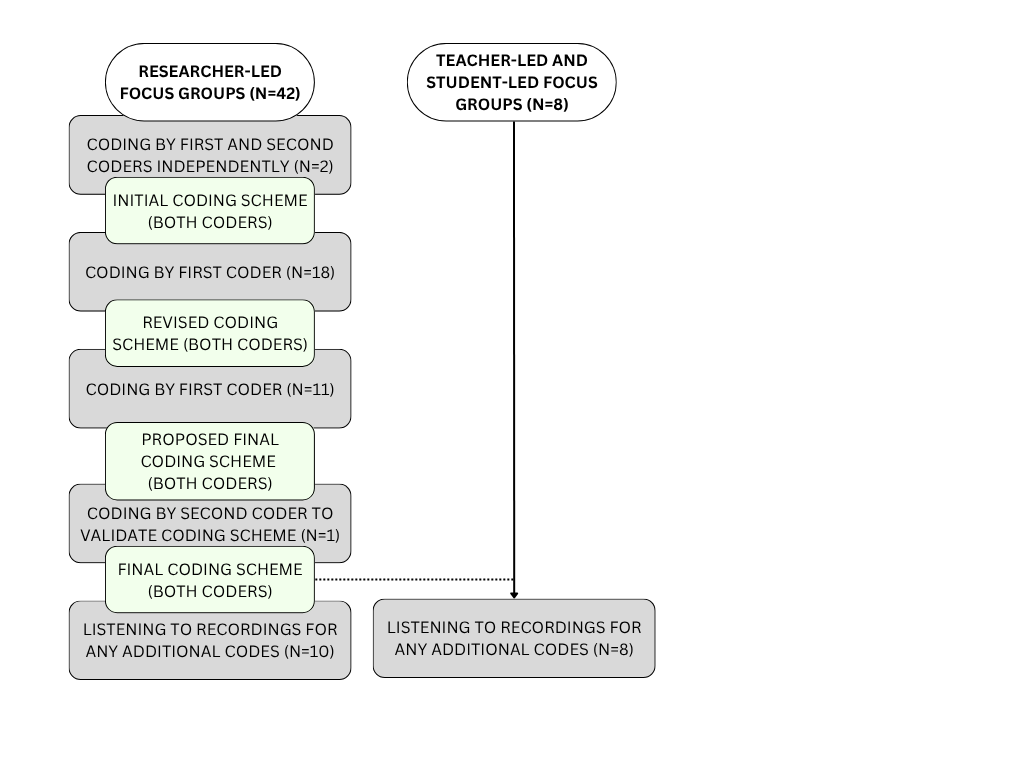


***Figure S4.*** Overview of the coding procedure of 50 focus groups with each 6-8 secondary school students.
